# A new model of the mechanism underlying lead poisoning: SNP in miRNA target region influence the AGT expression level

**DOI:** 10.1186/s41065-019-0084-x

**Published:** 2019-01-26

**Authors:** Yu Wu, Miaomiao Wang, Jinlong Zhang, Na Sun, Chunping Li

**Affiliations:** 1 Wuxi Medical School, Jiangnan University, 499 Jincheng Rd, Wuxi, 214023 People’s Republic of China; 2 Department of Occupational Health, Wuxi Center for Disease Control and Prevention, Wuxi, China

**Keywords:** Lead poisoning, 3′ untranslated region (3′ UTR), SNP, miRNA, AGT

## Abstract

**Background:**

To determine if the rs7079 polymorphism located in the 3′ UTR of the angiotensinogen gene (*AGT*) altered *AGT* gene expression and the risk of lead poisoning. A case-control study and luciferase reporter gene assay identified a significant association between rs7079 variants and the risk of lead poisoning.

**Results:**

Serum AGT levels were significantly higher in individuals carrying the rs7079 CA genotype, as compared to those carrying the rs7079 CC genotype. The binding of the miRNA mimics miR-31-5p and miR-584-5p to the 3′ UTR of *AGT* differed based on which rs7079 variant was present, implying that *AGT* gene expression depends on the rs7079 variant carried.

**Conclusions:**

The rs7079 C to A substitution reduced the binding of miR-31-5p/miR-584-5p to the 3′ UTR of *AGT*, possibly altering the risk of lead poisoning.

## Introduction

Lead is an important toxic agent that may be associated with population-level variations in cardiovascular disease rates [1]. Numerous population studies have shown that lead exposure can cause hypertension [2, 3]. However, the mechanisms involved in lead-induced hypertension remain to be clarified.

The renin-angiotensin-aldosterone system plays an important role in the regulation of blood pressure [4, 5]. Angiotensinogen (AGT), the initial substrate of the renin-angiotensin-aldosterone system pathway, is involved in the development of hypertension in humans and other animals [6, 7]. Animal studies have also shown that acute and chronic lead exposure cause hypertension and cardiovascular disease by altering the renin-angiotensin-aldosterone system: increasing angiotensin-converting enzyme activity [2, 8, 9], inhibiting Na^+^-K^+^-ATPase [10], inducing oxidative stress, reducing nitric oxide bioavailability, [11, 12] and depleting antioxidant reserves [13]. Many studies have provided new insights into the mechanisms by which lead can influence vascular function. Although these mechanisms have been proposed to explain lead-induced hypertension, its etiology remains unclear.

Polymorphisms in the *AGT* gene have significant effects on plasma AGT concentration, and are therefore involved in the pathogenesis of some diseases [6]. Su et al. [14] found that the rs7079 polymorphism was associated with blood pressure reduction in response to benazepril, while Ono et al. [15] showed that rs7079 might be a risk factor for non-alcoholic steatohepatitis. Al-Najai et al. [16] identified rs7079 as an independent risk factor for various deleterious cardiovascular traits. rs7079 has even been recognized as a factor in body fat distribution [17].

In addition, miRNAs often bind nucleotide sequences located in the 3′ Untranslated Region (UTR) of a given gene, modulating gene expression via post-transcriptional or post-translational mechanisms [18]. Because rs7079 is located on the 3’ UTR of the *AGT* gene, the *AGT* polymorphism might influence the binding of the miRNAs asiR-31 and miR-584 [19].

As lead exposure can increase blood pressure and *AGT* gene expression [2, 9], and the rs7079 polymorphism may affect *AGT* gene function, [19] it is possible that rs7079 may play a role in lead poisoning. However, the relationship between lead exposure and rs7079 has not previously been studied. Here, we hypothesized that the rs7079 variant in the *AGT* gene would be associated with lead poisoning. To test this hypothesis, we aimed to determine whether rs7079 might be associated with lead exposure in case-control study. We also aimed to determine whether the rs7079 polymorphism would influence the binding of the *AGT* 3′ UTR by miRNA.

## Materials and methods

### Study population

Our population-based case-control study included 304 individuals who had undergone a physical examination between 2012 and 2013 in Wuxi, China. Each participant completed a standardized questionnaire and signed a consent form. We drew 5 mL of blood from each participant, and used an atomic absorption spectrometer (AA800; Perkin-Elmer, Waltham, MA, USA) to detect blood lead levels (BLLs). BLLs were determined based on the National Occupational Health Standards of P. R. China, GBZ37–2002. Of the 304 participants, 114 individuals with blood lead levels (BLLs) ≥ 400 μg/L were considered lead poisoned (case group), while 190 individuals with BLLs < 200 μg/L were considered healthy (control group). The average lead concentration in production environment was 0.71 ± 0.43 mg/m^3^. Each individual in the case group reported at least 2 symptoms of lead toxicity, including headaches, nausea, gastritis, vomiting, lethargy, and poor appetite. Individuals who had smoked at least 1 cigarette per day for at least 1 year were defined as smokers, and individuals who consumed 3 or more alcoholic drinks per week for at least 1 year were considered drinkers [20]. All of our study protocols were approved by the Ethics Committee of Wuxi Center for Disease Control and Prevention.

### Genotyping

We extracted genomic DNA from peripheral blood lymphocytes of all samples. Extracted DNA was dissolved in TE buffer. We genotyped the *AGT* gene using the TaqMan method on a Roche LC 480 Real-Time PCR system (Roche Diagnostics, Shanghai, China). The primer and probe sequences used are available from the authors upon request. Negative controls were included on each plate to ensure the accuracy of the genotyping. Genotyping was performed blindly and independently by at least two different researchers. Approximately 10% of all samples were randomly selected for genotype confirmation; both sets of results were 100% concordant.

### Enzyme linked immunosorbent assay (ELISA)

We used a human AGT ELISA kit (Cusabio, Wuhan, China), which employs a quantitative sandwich enzyme immunoassay, to detect serum AGT levels in the case and control groups, following the manufacturer’s instructions. In brief, a microplate was pre-coated with an antibody specific to AGT. Standards and samples were pipetted into individual wells, such that all AGT was bound by the immobilized antibody. After removing any unbound substances, a biotin-conjugated antibody specific to AGT was added. After washing, we added avidin-conjugated horseradish peroxidase to the wells. Following another wash to remove any unbound avidin-enzyme reagent, a substrate solution was added to the wells, which developed color in proportion to the amount of AGT bound in the initial step. After color development stopped, we measured the intensity of the color.

### Plasmid construction and luciferase reporter assays

To construct luciferase reporter plasmids for the *AGT* 3′ UTR, we first amplified 613 bp fragments of the *AGT* 3′ UTR carrying the either the rs7079C or the rs7079A allele using PCR (forward primer: 5′- TCTAGGCGATCGCTCGAGGGCCAGGGCCCCAGAACAC -3′ and reverse primer: 5′- TATTGCGGCCAGCGGCCGCGGAGGCTTATTGTGGCAAGACG -3′). For cloning purposes, the forward primer carried an *Xho* I restriction site at the 5′-end, and the reverse primer carried a *Not* I restriction site at the 3′-end. The amplified products were treated with the restriction enzymes *Xho* I and *Not* I. Finally, the amplified fragments carrying either the C or A allele were inserted into several cloning sites of the PDS131_psiCHECK-2 reporter plasmid. The plasmids containing C or A allele was conducted, respectively. These insertions were confirmed by sequencing.

We used functional luciferase assays to determine whether the miRNA mimics miR-31-5p and miR-584-5p affected *AGT* gene expression, and whether changes in *AGT* gene expression were influenced by the rs7079 polymorphism. For the luciferase reporter assay, HEK293 cells were placed in 24-well plates (8 × 10^4^ cells per well). Half of the cells were co-transfected with PDS131_psiCHECK-2-7079C and psiCHECK-2, and half with PDS131_psiCHECK-2-7079A and psiCHECK-2 (both at a ratio of 50:1). The transfected cells were then re-transfected with miR-31-5p, miR-584-5p, or a non-targeting miRNA (negative control; GenePharma, Shanghai, China) at a final concentration of 20 nmol/μL.

To test the effect of lead on miRNA binding, transfected HEK293 cells were treated with 5 μM lead acetate during the luciferase assay. We measured luciferase activity in HEK293 cell lysates 48 h post transfection with a Dual-Luciferase Reporter Assay System (Promega, Madison, WI, USA), following the manufacturer’s instructions. Luciferase activity was normalized against firefly luciferase. Each plasmid construct was evaluated independently in triplicate.

### Statistical analyses

We used the χ^2^ test to evaluate differences among the frequency distributions of selected demographic variables, drinking status, and smoking status. We also used the χ^2^ test to compare the frequency distributions of each *AGT* allele and genotype between the case and control groups. We used multivariate logistic regressions to determine the adjusted odds ratios (ORs) and 95% confidence intervals (CIs). Two-sided tests of statistical significance were conducted using SAS (version 9.1; SAS Institute, Inc., Cary, NC, USA).

## Results

### Demographic characteristics of the study population

There were no significant differences between the case and control groups with respect to age, sex, drinking status, and smoking status (*P >* 0.05 for all; Table 1). The mean age of the case individuals was 41.9 (± 7.4 years; range: 22–60), and the mean age of the control individuals was 40.7 (± 5.3 years; range: 22–62). The average BLL in the case group (548.53 ± 109.82 μg/L) was significantly higher than that of the controls (59.80 ± 42.40 μg/L; *P* < 0.001). Individuals in the case group had significantly higher systolic (132.47 ± 11.25 mmHg) and diastolic (86.12 ± 11.32 mmHg) blood pressures than did the controls (122.68 ± 13.36 and 81.03 ± 10.32 mmHg, respectively; *P* < 0.001).

**Table 1:** Frequency distributions of selected variables in lead-exposed individuals and unexposed controls

Variables	BLL1^a^ (*n* = 190)		BLL2^b^ (*n* = 114)		*P* ^c^
	Samples	%	Samples	%	
Age (y)					0.103
≤ 39	76	40.0	35	30.7	
> 39	114	60.0	79	69.3	
Sex					0.062
Male	119	62.6	59	51.7	
Female	71	37.4	55	48.3	
Smoking status					0.126
Smoker	65	34.2	49	43.0	
Non-smoker	125	65.8	65	57.0	
Drinking status					0.411
Drinker	152	79.5	86	75.4	
Non-drinker	39	20.5	28	24.6	
BLL (μg/L)	59.80 ± 42.40		548.53 ± 109.82		< 0.001

^a^ Blood lead level (BLL) < 200 μg/L

^b^ Blood lead level (BLL) ≥ 400 μg/L

^c^ Two-sided χ^2^ test

### Genotypic distribution of the AGT polymorphisms

The rs7079 genotype frequencies in the control group were consistent with the Hardy-Weinberg equilibrium (*P* = 0.329; Table 2). The frequencies of the rs7079 polymorphisms CC, CA, and AA were significantly different between the control and case groups (*P* = 0.04 for all comparisons; Table 2). Moreover, a variant rs7079 genotype (CA or AA) appeared significantly more often in the case group (47.4%) than in the control group (33.3%; *P* = 0.02; Table 2). Thus, rs7079 variants CA and AA were associated with a significant risk of lead poisoning. Indeed, there was a significant association between individuals carrying either rs7079 CA or rs7079 AA and the risk of lead poisoning, relative to those carrying the rs7079 CC genotype (adjusted OR = 1.92, 95% CI = 1.16–3.18).

**Table 2:** Angiotensinogen gene (*AGT*) re7079 allele frequencies in the lead-exposed and unexposed (control) population, and the association of these polymorphisms with lead exposure

Genotypes	BLL1^a^ (*n* = 190)		BLL2^b^ (*n* = 114)		*P* ^c^	Crude OR (95% CI)	Adjusted OR (95% CI)^d^
	N	%	N	%			
rs7079							
CC	100	52.6	76	66.7	0.04	1.00	1.00
CA	88	46.3	36	31.6		1.86 (1.14–3.03)	2.27 (1.33–3.86)
AA	2	1.1	2	1.7		0.76 (0.11–5.52)	0.93 (0.12–7.31)
CA + AA	90	47.4	38	33.3	0.02	1.80 (1.11–2.92)	1.92 (1.16–3.18)
A Allele	92	24.2	92	17.5	0.08		

The observed genotype frequency among the control subjects was in agreement with Hardy-Weinberg equilibrium (*p*^2^ + 2*pq* + *q*^2^ = 1) *(χ*^*2*^ = 0.954, *P =* 0.329 for rs7079

^a^ Blood lead level (BLL) < 200 μg/L

^b^ Blood lead level (BLL) ≥ 400 μg/L

^c^ Two-sided χ^2^ test for either genotype or allele frequency

^d^ Obtained from logistic regression models adjusted for age, sex, and smoking habits

**Fig. 1:**
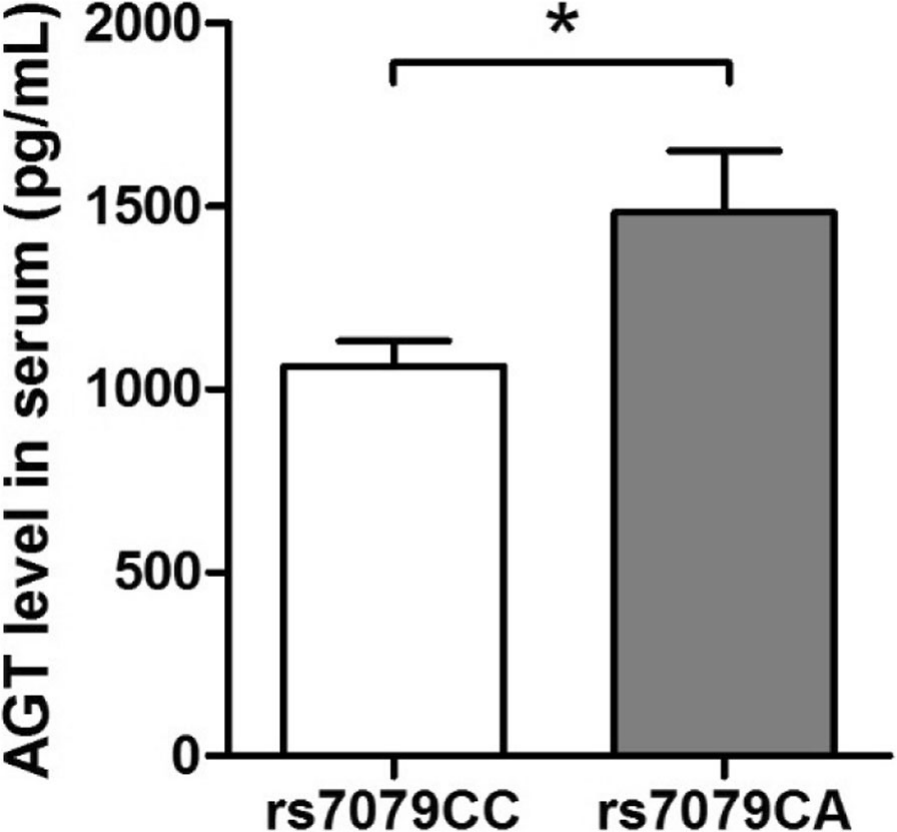
Difference in serum angiotensinogen (AGT) concentration in individuals with the *AGT* rs7079 CC genotype (*n* = 23) and those with the *AGT* rs7079 CA genotype (*n* = 16), measured with an enzyme linked immunosorbent assay (ELISA). Bars represent means ± standard deviations. *, *P* = 0.01

Our ELISA indicated that individuals carrying the rs7079 CC genotype had higher AGT serum concentrations than those carrying the CA genotype (*P* = 0.01; Fig. 1), suggesting that rs7079 was associated with AGT expression.

### The rs7079A allele reduced 3′ UTR binding by miRNA

**Fig. 2:**
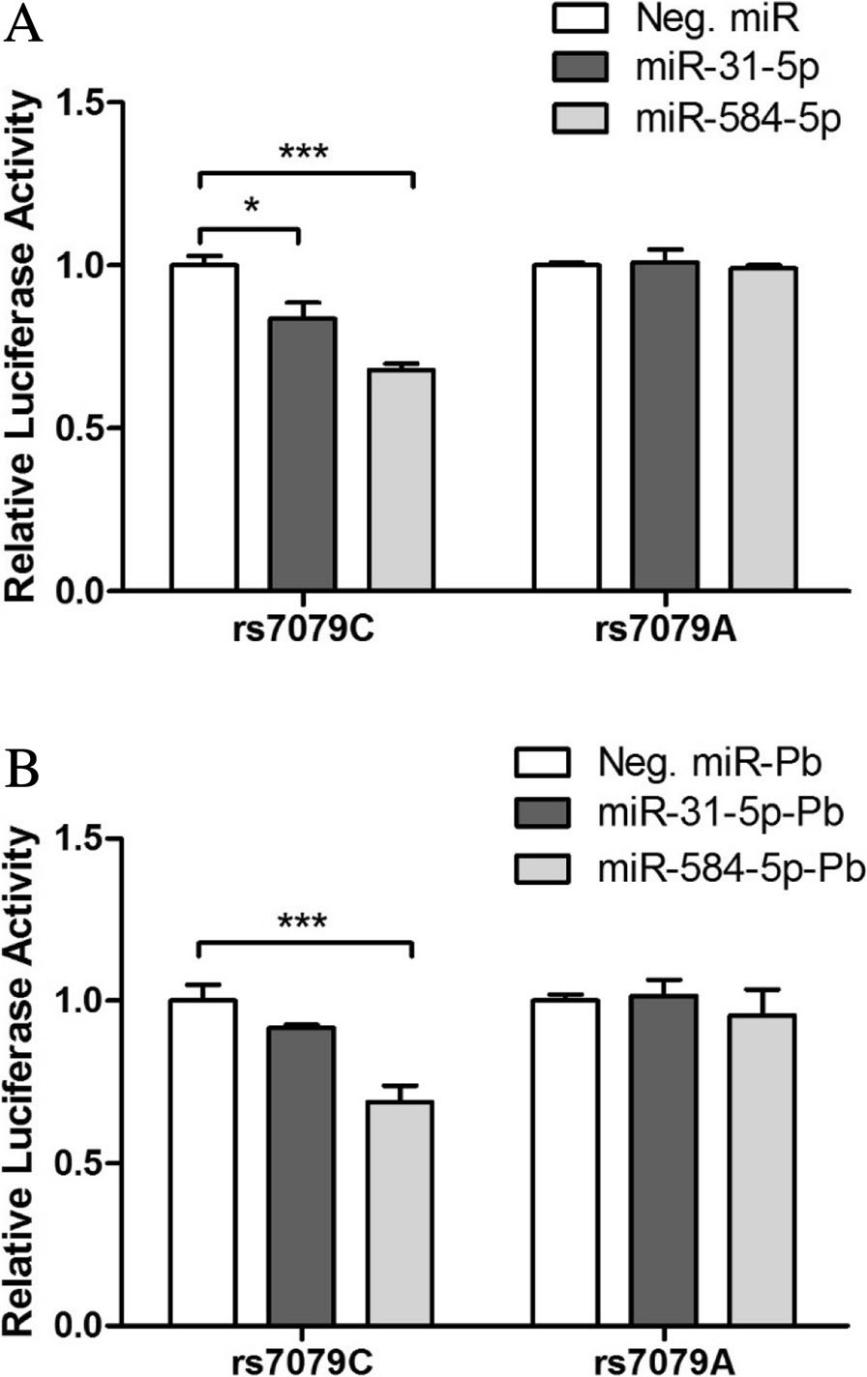
Results of dual luciferase assays where HEK293 cells were co-transfected with plasmid constructs carrying either the rs7079 C or the rs7079 A allele of the angiotensinogen gene (*AGT*), as well as either non-targeting miRNA (white bars; negative control), mRNA mimic miR-31-5p (black bars), or mRNA mimic miR-584-5p (gray bar). **a**: without lead. **b**: treated with 5 μM lead acetate. Luciferase activity is shown relative to the negative control. Bars represent means ± standard deviations of three independent transfection experiments. *, *P* = 0.01; ***, *P* < 0.001

Transfection with either miR-31-5p or miR-584-5p significantly suppressed luciferase expression in the presence of the rs7079C allele but not in the presence of the rs7079A allele (*P* < 0.05; Fig. 2a). The suppressive effect of miR-584-5p was greater than that of miR-31-5p. miRNA binding in the presence of the rs7079A allele was unaffected. This suggested that the rs7079A variant affected the binding of miRNAs to the 3′ UTR of the *AGT* gene, and might ultimately influence *AGT* transcription or translation.

### Lead had no direct effect on miRNA binding

Treatment with lead acetate slightly reduced the suppressive effect of miR-31-5p in the presence of rs7079C allele, but did not affect miR-584-5p. miRNA binding in the presence of the rs7079A allele was also unaffected.

## Discussion

Our major novel findings were as follows: 1) the *AGT* polymorphism rs7079 C was significantly associated with an increased risk of lead poisoning in a population from China; 2) the risk of lead poisoning was significantly higher in individuals carrying the *AGT* allele rs7079 A (genotypes rs7079 CA and rs7079 AA); 3) the presence of the *AGT* rs7079 A allele disrupted the binding of miR-31-5p and miR-584-5p to the 3′ UTR of *AGT,* and increased *AGT* gene expression, which is likely to reduce lead poisoning risk.

The epigenome may provide a suitable pathway by which lead exposure may influence disease susceptibility [21, 22]. Genetic analyses of polymorphisms in miRNA target regions have become increasingly common [23]. However, this is the first study to examine the association between SNPs in the miRNA target region of the *AGT* gene and lead poisoning. Our results increase our understanding of the mechanisms by which *AGT* is involved in lead poisoning.

Lead exposure increases the risk of developing hypertension and other cardiovascular diseases [24]. AGT is an important regulator of blood pressure, and SNPs in the *AGT* gene are associated with increases in serum AGT levels and hypertension [6, 25–28]. In a previous study, we found that lead exposure increased blood pressure by increasing serum AGT [29]. In this study, we found the blood pressure in cases was higher than controls, although it had not arrived the level of hypertension. It might own to the “health worker effect” [30]. We found no direct association between blood pressure and rs7079 variant, however, it might indicate the complexity of mechanisms of lead poisoning,

Gene SNPs can be used to predict the toxicity of lead exposure [31, 32]. Here, rs7079 was significantly associated with the risk of lead poisoning, and individuals carrying the rs7079 A allele were at an increased risk of lead poisoning. Serum AGT concentrations were lower in individuals carrying the rs7079 C allele than in those carrying the rs7079 A allele, possibly because the rs7079 A allele decreases the binding between miRNAs and the *AGT* 3′ UTR. Therefore, our results suggested that rs7079 is a functional SNP, consistent with previous studies indicating that rs7079 was associated with non-alcoholic steatohepatitis [15], body fat distribution [17], and coronary artery disease [16].

Consistent with our results, a previous study used a luciferase reporter gene assay to show that the rs7079 A allele altered the binding of two miRNA mimics (miR-31-5p and miR-584-5p) to the 3′ UTR of *AGT* [19]. However, miRNA function was not directly affected by lead exposure.

Our study had some specific limitations. First, sample sizes were small, particularly for the lead poisoning group, and more samples may serve to clarify our results. Second, due to the lack of samples, it was impossible to identify the association between the *AGT* SNP and miRNA binding in the lead-poisoned group. Third, AGT activity detection might be more convictive. Future work with larger sample sizes should focus on the function of rs7079 to clarify its specific involvement in lead poisoning.

## Conclusions

This population-based case-control study indicated that the presence of the rs7079 A allele in 3′ UTR of the *AGT* gene significantly increased the risk of lead poisoning. The rs7079 C to A substitution weakened the binding between miR-31-5p/miR-584-5p and the 3′ UTR of *AGT,* possibly increasing *AGT* expression and, consequently, altering the risk of lead poisoning.

## Abbreviations

3′ UTR: 3′ untranslated region
AGT: Angiotensinogen
BLLs: Blood lead levels
CI: Confidence interval
ELISA: Enzyme linked immunosorbent assay
OR: Odds ratio
PCR: Polymerase chain reaction
SNP: Single nucleotide polymorphism
